# Cost of a community mental health service: a retrospective study on a psychosocial care center for alcohol and drug users in São Paulo

**DOI:** 10.1590/1516-3180.2018.0164310818

**Published:** 2018-10-22

**Authors:** Paula Becker, Denise Razzouk

**Affiliations:** I MSc. Occupational Therapist and Doctoral Student, Centro de Economia em Saúde Mental (CESM), Department of Psychiatry, Universidade Federal de São Paulo (UNIFESP), São Paulo (SP), Brazil.; II PhD. Psychiatrist and Affiliated Professor, Centro de Economia em Saúde Mental (CESM), Department of Psychiatry, Universidade Federal de São Paulo (UNIFESP), São Paulo (SP), Brazil.

**Keywords:** Substance-related disorders, Community mental health services, Costs and cost analysis, Direct service costs, Substance abuse treatment centers

## Abstract

**BACKGROUND:**

Psychosocial care centers for alcohol and drug users (CAPS-ad) are reference services for treatment of drug users within the Brazilian National Health System. Knowledge of their total costs within the evidence-based decision-making process for public-resource allocation is essential. The aims here were to estimate the total costs of a CAPS-ad and the costs of packages of care (according to intensity of care); to ascertain the ratio between total CAPS-ad costs and the federal funding allocated; and to describe the methods for estimating unit costs for each CAPS-ad cost component.

**DESIGN AND SETTING:**

Retrospective study conducted in a public community mental health service.

**METHODS:**

This was a retrospective cost description study on a CAPS-ad located in a city in the state of São Paulo, using a public healthcare provider perspective and a top-down approach, conducted over a 180-day period from March 1 to August 30, 2015.

**RESULTS:**

The total mean monthly costs of the CAPS-ad were BRL 64,017.54. Healthcare staff accounted for 56.5% of total costs. The mean costs per capita and per month for intensive and non-intensive care packages were, respectively, BRL 668.34 and BRL 37.12.

**CONCLUSIONS:**

The federal budget allocation covered 62.1% of the CAPS-ad costs and the remaining 37.9% end up funded by the municipal government. The cost of the intensive package of care was 18 times greater than the non-intensive package. Developing criteria for using services and different packages of care based on patients’ needs, and optimizing human resources according to specific actions, may improve people’s mental health and avoid wasted resources.

## INTRODUCTION

Substance-related disorders have been a priority of the Brazilian public health agenda since the beginning of the last decade, when the federal government established specific policies and programs,^1^ such as the Psychosocial Care Network (RAPS). This priority agenda is based on the prevalence of drug and alcohol use in Brazil, and on their consequences for users and society.^2^^,^^3^^,^^4^ The main care strategy for treating substance-related disorders within RAPS comprises community-based mental health services (CMHS), known as psychosocial care centers for alcohol and drug users (CAPS-ad). Knowledge of their costs within the decision-making process for allocation of public resources is essential.

Brazil is a country of huge territorial extent, but 50% of the Brazilian population is concentrated in only 5% of its municipalities.^1^ This characteristic has justified regionalization of this country’s healthcare management, and the three levels of government (federation, states and municipalities) are expected to agree on the management and costing of healthcare services. The CAPS-ad system is partially funded by the national government, but so far there is no information regarding whether the federal funding entirely covers the costs of CAPS-ad, and what the budget impact on municipalities would be, in relation to implementing and maintaining these services.

The territorial complexities of Brazil, along with the need to expand mental health care coverage for people with substance-related disorders, highlight the need for economic planning through cost studies and economic evaluations.^5^ Very few economic studies have examined psychosocial interventions for people with substance-related disorders in low and middle-income countries.^6^ This was corroborated by our finding from the literature, while conducting this study, that no cost studies on CAPS-ad have been undertaken. Data on CAPS-ad costs would provide support for the decision-making process regarding opening new services of this nature, through helping healthcare managers to analyze the feasibility of maintaining such services over time.

The main aim of this study was to estimate the total costs of a CAPS-ad that is located in a city in the state of São Paulo, and the costs of three packages of care (intensive, semi-intensive and non-intensive) delivered by this service for patients with substance-related disorders, from the public healthcare provider perspective. The additional objectives were to ascertain the ratio between total CAPS costs and the federal funding allocated to this service and to describe the methods for estimating the unit costs for each CAPS cost component.

## METHODS

This was a cross-sectional study that was conducted to estimate the total costs of a CAPS-ad located in the city of Rio Claro, state of São Paulo, covering a 180-day period from March 1 to August 30, 2015. Data on costs were extracted from the municipal accounting database and a top-down approach was applied for the cost estimation. The CAPS-ad costs were estimated for a 180-day period in the year 2015 and then the ratio between costs funded by the federal and municipal governments was examined. The 180-day period was chosen because this provided the best data quality in the administrative database that was made available by the municipal government. This study adopted the perspective of the public healthcare provider because there are no national or regional published data on the unit costs and cost components of this kind of service in Brazil.

This study was approved by the Research Ethics Committee of the Federal University of São Paulo (Universidade Federal de São Paulo, UNIFESP), under protocol number 0296/15.

### Service description: psychosocial care center for alcohol and drug users (CAPS-ad)

A CAPS-ad is a community-based mental health service that promotes public comprehensive care for people with substance-related disorder. The CAPS-ad that was studied here covers a population of approximately 215,960 people. It welcomes spontaneous and referred demand, is integrated with primary care and psychiatric and general hospitals for acute inpatient hospitalizations. This service is one of five that, together, make up the municipal mental health setting: another two CAPS, of which one deals with all mental disorders and is open 24 hours per day and the other deals with children and adolescents; and two outpatient services. The service that is the focus of the present study offers treatment through three types of package of care: intensive, semi-intensive and non-intensive.

Individuals with severe use of drugs are generally directed towards the intensive package of care. The semi-intensive package of care is for individuals who seek the service presenting moderate use of drugs that puts them at risk of greater worsening of their functioning; or those who migrate to an intermediate treatment after showing improvements through intensive treatment. The non-intensive package of care offers support for users who have good social and family ties, those who make moderate or severe use of drugs but who work and can only attend the service at specific times, or those who are in the process of leaving the service because they have presented improvements.

### Estimation of cost components

The following CAPS-ad cost component categories were considered:

*Healthcare staff costs*: These were the costs of healthcare professionals working at this center, including two psychiatrists, one general practitioner, one nurse, two nursing technicians, two psychologists, two occupational therapists and one social worker. Firstly, their total costs over a 180-day period were estimated. Secondly, unit costs were determined. Lastly, the costs for each healthcare staff intervention within the care packages were estimated.

*Medication costs:* These were categorized as the costs of psychotropic and non-psychotropic medications. Firstly, the per capita monthly costs of medication consumption were obtained. Secondly, the per capita monthly costs were extrapolated to the 180-day period, considering the mean number of patients assisted by the service over this period (810 patients). Then, the unit costs were defined.

*Revenue costs*: These were the costs of support services (diet service: lunch and snacks), utilities (expenses with electricity, telephone and gas consumption), consumables (medical supplies, catering, stationery and cleaning supplies), non-healthcare human resources (security, which was provided through an outsourced hired service hired; and cleaning services, consisting of one cleaning professional) and overheads (healthcare manager, one assistant and one receptionist). Their costs were estimated considering the number of working hours and salaries.

*Capital costs:* These consisted of rent, equipment and building repair. Equipment costs were adjusted according to the consumer price index by using the market price for 2015 (presented as Supplementary Table S1). The unit costs were extracted from three online stores in September 2015. Then, these costs were annuitized by estimating the equivalent annual annuity (EAA), with a 5% discount factor (standard in Brazil), and by taking the lifetime use of equipment to be five years, as can be seen in Drummond et al*.*^7^

### CAPS-ad funding sources

**Figure 1.**
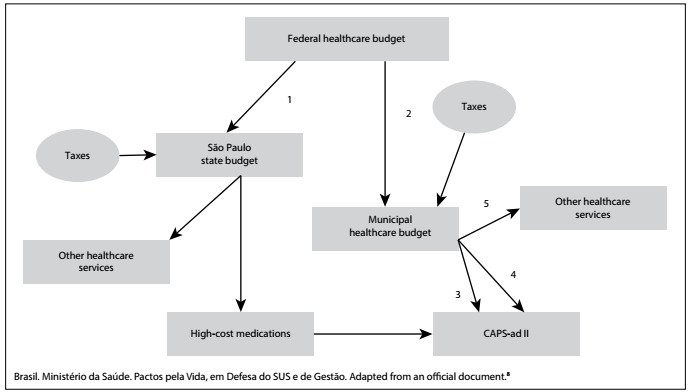
Funding sources for Psychosocial Care Centers for Alcohol and Drug Users in Brazil.

It is expected that CAPS-ad will be funded from federal, state and municipal public healthcare budgets (Figure 1). The federal healthcare budget is allocated to the state and municipal governments through six funding packages, of which two are oriented towards CAPS-ad: the *Medication* funding package (arrow 1) and the *Medium and High-Complexity* healthcare funding package (arrow 2).^8^ The *Medication* funding package is firstly allocated to the state government for drug purchasing.

The *Medium and High Complexity* healthcare funding is allocated directly to the municipal healthcare budget to fund CAPS-ad and other healthcare services. In 2015, the federal government released Brazilian reais (BRL) 39,780.00 per month to fund each CAPS-ad within the national territory, and the municipal healthcare budget funded the remaining costs, which were not publicly known until the analysis of the present study. In Figure 1, arrows 3 and 4 respectively represent the federal government budget allocated to municipal government and the municipality’s own healthcare budget, which were both used to fund CAPS-ad.

### Cost components: packages of care

The *intensive package of care* (IPC) offered support from Monday to Friday, from 8 am to 5 pm in this CAP-ad. This one-month package included: one visit to a psychiatrist, one visit to a general practitioner, 12 group sessions with an occupational therapist, 12 group sessions with a psychologist, 10 group sessions with a social worker, six group sessions with a nurse, 20 individual routine nursing care sessions and eight individual sessions with a health case manager, approximately. One visit to a psychiatrist or general practitioner was estimated to last for 30 minutes. The group sessions were considered to last 90 minutes and each session was expected to cater for a mean of 10 patients per session. An individual session was estimated to last for 60 minutes, and an individual routine care session with an assistant nurse care was estimated to last for 15 minutes. The *health case manager* could be an occupational therapist, a nurse, a social worker or a psychologist, and their individual sessions were estimated to last for 60 minutes.

The *semi-intensive package of care* (SIPC) was delivered on three days a week. This one-month package included: one visit to a psychiatrist, one visit to a general practitioner, eight group sessions with an occupational therapist, eight group sessions with a psychologist, four group sessions with a social worker, four group sessions with a nurse, 12 individual routine care sessions with a nurse and four individual sessions with a health case manager, following the same patterns of length as mentioned previously.

Patients attending the IPC and the SIPC sessions received three meals per day during the treatment: breakfast, lunch and afternoon snacks.

The one-month *non-intensive package of care* (NIPC) included one visit to a psychiatrist and one weekly group session with a health case manager. Meals were not included in this package.

### Analyses

Descriptive analyses on cost distributions were carried out for each cost component. Unit costs were calculated by means of a top-down approach, and unit costs for each mental health intervention per individual were estimated, considering the average length of one visit and one-tenth of the costs of a group session. The ratio between funding sources was obtained by calculating the cost difference between the total CAPS costs and the federal government funding. Sensitivity analysis was carried out both on the total costs of the CAPS-ad and on the total costs of the packages of care, in order to enable transferability of the study results to other Brazilian regions. Regarding the total costs of the CAPS-ad, a 15% cost variation in healthcare staff costs (rate established in accordance with the mean salary variation among the Brazilian regions in relation to the average salary of workers in the southeastern region)^9^ and a 30% variation in the costs of rent,^10^ revenue, medications and maintenance/repairs upwards (worst scenario) and downwards (best scenario) were considered. Lastly, in relation to the packages of care, two distinct scenarios were considered for the group sessions, with five participants (worst scenario) and 15 participants (best scenario) per session, especially because there are large variations in the numbers of participants in group sessions in the real context of mental health services.

## RESULTS

Table 1 shows the unit costs per mental health professional and the total cost per professional category for the 180-day period. The healthcare staff cost was BRL 216,918.00 over this period, which was equivalent to USD 68,213.20 after conversion using purchasing parity power (PPP) exchange rates relating to 2015.^11^

**Table 1.** Total costs and unit costs relating to healthcare staff in Brazilian reais (BRL)*

Cost components	Quantity	Worked hours over the 180-day period	Total costs in 180 days over the 180-day period, in BRL	Unit cost	Cost per unit BRL
Healthcare staff					
Psychiatrist	2	960	54,069.48	Hour	56.32
General practitioner	1	480	27,034.74	Hour	56.32
Occupational therapist	2	1,440	35,974.32	Hour	24.98
Psychologist	2	1,920	35,974.32	Hour	18.73
Social worker	1	720	19,040.70	Hour	26.44
Nurse	1	960	19,040.70	Hour	19.83
Assistant nurse	2	1,920	25,784.40	Hour	13.42
Total	11	8,400	216,918.66	-	-

*1 USD (United States dollars) = BRL 3.18 (using purchasing parity power exchange rate for 2015).^9^

The mental health treatments offered by the CAPS-ad and their respective costs are described in Table 2.

**Table 2.** Unit costs for mental health treatment in Brazilian reais (BRL)

Healthcare staff	Description	Unit cost	Cost per capita, 2015
Visit to psychiatrist	Individual visit of 30 min	Visit	28.16
Visit to general practitioner	Individual visit of 30 min	Visit	28.16
Group session with occupational therapist	90-min group session, with an average of 10 patients	Session	3.74*
Group session with social worker	90-min group session, with an average of 10 patients	Session	3.96*
Group session with psychologist	90-min group session, with an average of 10 patients	Session	2.80*
Group session with nurse	90-min group session, with an average of 10 patients	Session	2.97*
Individual session with assistant nurse	15-min individual appointment	Appointment	3.35
Individual session with health case manager	60-min individual appointment	Appointment	22.49
Group session with health case manager	90-min group session, with an average of 10 patients	Session	2.24*

*Cost of one group session per patient.

Table 3 shows the total revenue and total medication costs and their unit costs. The total revenue and total medication costs over the 180-day period were, respectively, BRL 93,425.72 (USD 29,379.15, using PPP) and BRL 66,058.04 (USD 20,772.96, using PPP).

**Table 3.** Total revenue and total medication costs in Brazilian reais (BRL)

Cost components	Total costs over 180-day period	Minimum cost per month	Maximum cost per month	Unit cost	Cost per unit
Revenue costs					
Support Services					
Diet - lunch	23,330.60	3,457.00	4,462.70	Per lunch	11.00
Diet - snacks	379.07	46.80	141.05	Per snack	0.23
Total	23,709.67	3,503.80	4,603.75	-	-
Non-health human resources					
Cleaner	9,129.42	-	-	Hour	9.50
Security	2.454.36	-	-	Hour	2.55
Total	11,583.78	1,930.63	1,930.63	-	-
Utilities					
Electricity	1,420.94	185.94	236.82	Day	7.89
Telephone	1,977.44	298.51	329.57	Day	11.00
Gas	47.00	0	47.00	Day	0.26
Total	3,445.38	484.45	613.39	-	-
Consumables	5,019.59	572.54	836.54	Day	27.89
Total	5,019.59	572.54	836.54	-	-
Overhead					
Healthcare manager	29,489.00	-	-	Hour	15.36
Receptionist	9,129.42	-	-	Hour	4.75
Secretary	11,048.88	-	-	Hour	5.75
Total	49,667.30	8,277.88	8,277.88	-	-
Total	93,425.72	14,769.30	16,262.19	-	-
Medication costs					
Psychotropic	46,209.80	-	-	Day	256.72
Non-psychotropic	19,848.24	-	-	Day	110.26
Total	66,058.04	-	-		

Regarding capital costs over the 180-day period, the rent cost was BRL 6,826.68, or BRL 37.92 per day, and the maintenance/repair cost was BRL 125.50, or BRL 0.70 per day. The total cost of equipment was BRL 6,500.00, or BRL 750.67 over the 180-day period and BRL 4.17 per day. The total capital cost over the 180-day period was BRL 7,702.85 (USD 2,422.27, using PPP).

Considering healthcare staff, revenue, medications and capital costs, the CAPS-ad costs over the 180-day period were BRL 384,105.27, i.e. an average of BRL 64,017.54 or USD 20,131.54 (using PPP) per month. Healthcare staff accounted for 56.5% of the total CAPS-ad costs, revenue costs 24.3%, medication costs 17.2% and capital costs 2%. Table 4 shows the sensitivity analysis results on the total CAPS-ad costs in the best and worst scenarios, compared with the total costs for the service.

**Table 4.** Sensitivity analysis for total CAPS-ad costs in Brazilian reais (BRL)

	Base Cost	Best scenario				Worst scenario			
		(%)	Variation (BRL)	Cost (BRL)	Variation/base cost (BRL)	(%)	Variation (BRL)	Cost (BRL)	Variation/base cost (BRL)
Healthcare staff costs	216,918.66	-15.0	-32,537.80	184,380.86		+15.0	+32,537.80	249,456.46	
Revenue costs	93,425.72	-30.0	-28,027.72	65,398.00		+30.0	+28,027.72	121,453.44	
Medication costs	66,058.04	-30.0	-19,817.41	46,240.63		+30.0	+19,817.41	85,875.67	
Capital cost	7,702.85			7,342.00				7,582.57	
Equipment	6,500.00	5.0		6,500.00		5.0		6,500.00	
Rent	1,000.00	-30.0	-300.00	700.00		+30.0	+300.00	1,300.00	
Maintenance/repair	202.85	-30.0	-60.86	142.00		+30.0	+60.86	263.71	
Total	384,105.27			303,361.49	-21.02%			464,368.14	+20.89%

In the same year, the federal funding allocated to the municipal government for financing the CAPS-ad was BRL 39,780.00 per month or BRL 238,680.00 (USD 75,056.60, using PPP) over the 180-day period.^12^ In other words, federal funding covered 62.1% of the total CAPS-ad cost, and the municipal government funded the remaining 37.9%, or BRL 145,425.27 (USD 45,731.21, using PPP) over the 180-day period, and BRL 24,237.54 per month (USD 7,621.86, using PPP).

Over the 180-day period, the CAPS-ad offered assistance to approximately 810 patients. Thus, the mean monthly CAPS-ad treatment cost per capita was BRL 474.20 (USD 149.12, using PPP). BRL 294.47 was funded by the federal government and BRL 179.73 by the municipal government.

Data on the care packages are shown in Table 5. Based on the unit costs for mental health treatment at this CAPS-ad (Table 2), it was possible to estimate the cost of a one-month package of care per capita. Monthly, the IPC cost BRL 668.34 (USD 210.16, using PPP), the SIPC cost BRL 404.04 (USD 127.05) and the NIPC cost BRL 37.12 (USD 11.67). Considering the best scenario, with 15 participants per group session, the per capita cost was reduced by approximately 33%, i.e. the group session with an occupational therapist with 10 participants had a per capita cost of BRL 3.74 and, with 15 participants, the value dropped to BRL 2.50. In the worst scenario, in which there were only five participants per group session, the per capita cost was raised by approximately 100%, i.e. the per capita cost of the group session with a social worker rose to BRL 7.93, and with a psychologist to BRL 5.62. Overall, in the worst scenario, the IPC would be 20.4% more expensive, and in the best scenario, 6.7% cheaper.

**Table 5.** One-month package-of-care costs per capita in Brazilian reais (BRL)

Mental health treatment	IPC		SIPC		NIPC	
	Quantity	Total cost	Quantity	Total cost	Quantity	Total cost
Visit to psychiatrist	1	28.16	1	28.16	1	28.16
Visit to general practitioner	1	28.16	1	28.16	0	0
Group session with occupational therapist	12	44.88	8	29.92	0	0
Group session with social worker	10	39.60	4	15.84	0	0
Group session with psychologist	12	33.60	8	22.40	0	0
Group session with nurse	6	17.82	4	11.88	0	0
Individual session with assistant nurse	20	67.00	12	40.20	0	0
Individual session with health case manager	8	179.92	4	89.96	0	0
Group session with health case manager	-	-	-	-	4	8.96
Lunch	20	220.00	12	132.00	0	0
Snacks	40	9.20	24	5.52	0	0
Total cost per month		668.34		404.04		37.12

IPC = intensive package of care; SIPC = semi-intensive package of care; NIPC = non-intensive package of care.

## DISCUSSION

The Brazilian federal government spent BRL 39,780.00 per CAPS-ad in 2015,^12^ and the present study showed that the mean monthly cost of this service was BRL 64,017.54 in the same year, i.e. federal funding covered 62.1% of the total costs of the CAPS-ad examined here. This CAPS-ad offered treatment for an average of 135 patients per month and, according to the federal-to-municipal government funding ratio, the federal government invested BRL 294.47 (USD 92.60, using PPP) and the municipal government BRL 179.73 (USD 56.51, using PPP) for each patient assisted. To use this resource as effectively as possible, it is necessary to ascertain strict eligibility criteria for treatment proposals, especially when considering the huge difference in costs between the packages of care.

Healthcare staff accounted for 56.5% of the total CAPS-ad cost. This result was close to the findings of Araujo et al.,^13^ who showed that the expenditure on human resource payments represented 75.9% of all Brazilian municipal governments’ expenditure, regarding all healthcare expenses. Moreover, another cost analysis study by Razzouk et al.,^14^ on residential facilities in the city of São Paulo, showed that human resources accounted for 61.4% of its total costs. Even though the CAPS-ad of the present study may not be representative of all Brazilian CAPS-ad in some respects, its total estimated costs may be considered comparable to those of other CAPS-ad given that it followed the standard regulations regarding healthcare staff composition,^15^ which represents the greatest part of the costs.

The Rio Claro municipal government funded 37.9% of the monthly CAPS-ad costs, or BRL 64,017.54. In that same year, the total budget of the city council was BRL 698 million, and 30% (BRL 209 million) was invested in healthcare according to the official city council newspaper.^15^ Thus, 0.36% of the total municipal healthcare budget was invested in the only specialized service for psychosocial rehabilitation that assisted drug users in the city.

Although the federal government’s budget covered a large part of the CAPS-ad costs, there have been no adjustments for inflation since 2011,^12^ regarding the resources allocated to municipal governments for financing CAPS-ad, which may have made these resources less significant over time. Therefore, given that the resource allocation from the federal to the municipal government for funding specific services is insufficient, municipal managers have become obliged to increase their contribution towards maintenance of these services.^13^ Thus, municipalities have become the Brazilian governmental level that contributes most to the healthcare sector in proportional terms, i.e. in relation to its tax collection.^16^^,^^17^

The monthly CAPS-ad treatment cost per capita was almost three times lower than the amount paid per month (BRL 1,350.00) by the São Paulo state government to provide inpatient treatment for substance users at private clinics through the “restart program” (Programa Recomeço).^18^ It is important to underscore that the decision-making process regarding public resource allocation between different services should be based not only on costs but also on patients’ needs and profiles. Moreover, the differences in costs mentioned above were not compared with differences in outcomes or according to sample characteristics. However, it is also important to establish strict criteria for using more expensive services, in accordance with patients’ needs, in order to optimize the public resource allocation.

The unit costs reported here may inform further economic evaluations and modelling studies within similar contexts of services in the state of São Paulo. This is especially important, considering the lack of information on unit costs within healthcare in Brazil. This is unlike the situation in some European countries, where national guidelines for unit costs reference for healthcare services and interventions are available, thus facilitating development of cost studies and economic evaluations.^19^^,^^20^ There is a lack of cost-effectiveness studies in Brazil, especially in relation to mental health, and the findings from the present study may be useful for further studies in this regard.

The expansion of the CAPS network in Brazil^1^ and the way in which public resource allocation for funding a CAPS-ad occurs^8^^,^^15^ show that only at the federal level is there any specific budget for this purpose. A specific budget for mental health actions has been placed as a priority for mental health policies worldwide,^21^^-^^22^ and Brazilian states and municipalities need to ensure transparency in the way that they apply resources for mental health services.

According to the current legislation on public health financing in Brazil,^23^^,^^24^ municipalities and states should, respectively, allocate minimums of 15% and 12% of their budgets to public health. However, state governments’ participation in the cost of CAPS in Brazil remains a challenge and has been the subject of interpellation instigated by municipalities, with the aim of achieving greater participation from state governments, so as to ensure sustainability of these services.^1^

It is necessary to ascertain municipal governments’ capacity to manage the costs of these services, in order to plan public investment in services that can be sustained over time. After the costs of this type of service have been established, the discussion can shift from focusing on coverage to analysis in greater depth, including in relation to the cost-effectiveness of services and interventions.

The present study has three important limitations:

1. the state government may have participated in the purchase of medications prescribed by CAPS-ad doctors, and thus its participation in the CAPS-ad funding system may have been underestimated;
2. the study period may not have detected possible fluctuations in costs over the twelve months of the year; and
3. there is some uncertainty regarding inaccuracies of cost estimations, given the variations in healthcare staff salaries, revenue and medication costs according to the different regions of Brazil. These limitations impede generalizations.

## CONCLUSIONS

The federal government funded two-thirds of the CAPS-ad costs, while one-third was funded by the municipal government. These findings may enable better planning and management, both for the federal government and for municipal governments that are interested in expanding the CMHS network for people with substance-related disorders. Moreover, these findings also highlight the need for government agencies and the national academic community to focus on mental health policies, not only to expand treatment coverage, but also to attain the best allocation of resources, in terms of costs and outcomes. Careful use of packages of care based on patient needs can improve people’s mental health and avoid wasting resources.

## Supplementary File.

**Table S1.** Description and estimation of CAPS-ad equipment costs according to website consultation in September 2015

Equipment	Quantity	Brand and model	Year of purchase	Websites consulted	Cost per unit (R$)
Desktop computer	4	Lenovo V520s, Intel Core I3, 2 GB, 500 GB	2012	americanas.com.br magazineluiza.com.br submarino.com.br	800.00
Computer monitor	4	LG Monitor 20M37AA	2012	americanas.com.br magazineluiza.com.br walmart.com.br	340.00
Printer	1	HP Deskjet Multifunction (p2035)	Before 2010	americanas.com.br magazineluiza.com.br walmart.com.br	499.00
Refrigerator	1	Refrigerator with one door, Consul	Before 2010	casasbahia.com.br magazineluiza.com.br extra.com.br	870.00
Stove	1	Four-burner stove, Consul	Before 2010	casasbahia.com.br magazineluiza.com.br extra.com.br	570.00
